# The Relationship Between Successful Aging and All-Cause Mortality Risk in Older Adults: A Systematic Review and Meta-Analysis of Cohort Studies

**DOI:** 10.3389/fmed.2021.740559

**Published:** 2022-02-09

**Authors:** Lifen Mao, Rulan Yin, Jianzheng Cai, Mei'e Niu, Lan Xu, Wenjie Sui, Xiaoqing Shi

**Affiliations:** ^1^Department of Nursing, The First Affiliated Hospital of Soochow University, Suzhou, China; ^2^Department of Rheumatology, The First Affiliated Hospital of Soochow University, Suzhou, China; ^3^Faculty of Nursing, Chiang Mai University, Chiang Mai, Thailand

**Keywords:** successful aging, healthy aging, mortality, older adults, meta-analysis

## Abstract

**Background:**

This meta-analysis aimed to explore the effect of successful aging (SA) on all-cause mortality risk in older people to provide a theoretical basis for promoting SA.

**Methods:**

PubMed, Embase, CINAHL, CNKI, and WanFang databases (inception to March 4, 2021) were searched for cohort studies to evaluate the relationship between SA and mortality in older people. A random-effects model was used to synthesis hazard ratio and 95% confidence intervals. Quality assessment was performed using the Newcastle–Ottawa scale. All statistical analyses were conducted in STATA 16.0.

**Results:**

In total, 21,158 older adults from 10 studies were included in the current systematic review and meta-analysis. The SA group tended to have 50% lower risk of all-cause mortality than the non-SA group (pooled hazard ratio = 0.50, 95% confidence intervals: 0.35–0.65, *P* < 0.001; *I*^2^ = 58.3%). The risk of all-cause mortality in older people increased by 17% for each unit increment in the healthy aging index (HAI) (*I*^2^ = 0%, *P* = 0.964). Compared with the reference group (HAI 0-2), older people with HAI 3-4, HAI 5-6, and HAI 7-10 had 1.31-fold, 1.73-fold, and 2.58-fold greater risk of all-cause mortality, respectively. Subgroup analysis did not reveal possible sources of heterogeneity.

**Conclusions:**

This meta-analysis suggests that older adults with SA reduced the risk of all-cause mortality by 50%. However, few interventional studies have been conducted. Therefore, healthcare providers must be aware of the relationship between SA and mortality risk and actively develop intervention methods for helping old people achieve SA.

## Introduction

With the development of medical technology and increasing interest in health, there has been a gradual growth in average life expectancy (1) as well as a continuous decline in birth rate, making aging one of the major public health challenges in the world (2). It is expected that the world population will increase by 2 billion in the next 30 years from 7.7 billion currently to 9.7 billion in 2050. In 2019, 9% of the world population was over age 65, and by 2050, it will be 16% (3). Aging is saturated by feelings of loneliness and dependence on others, along with degenerated health and physical and cognitive functions (4). However, some individuals age successfully without severe disease or disability, enjoying high cognitive, physical, and social functioning (5–7). Therefore, great efforts must be made to achieve successful aging (SA) among older populations to reduce the negative impact of aging (8).

The concept of SA has evolved in various directions. According to the classical definition by Rowe and Kahn (5), SA refers to a later life with less disease and disease-related disability, high level of cognitive and physical functions, and an active lifestyle. The notion of SA reveals an important perspective to understand later life by which aging is no longer characterized by an inevitable and irreversible life stage with morbidity, but can instead be viewed as a process full of possibilities to recirculate and enhance physical, cognitive, emotional, and social functioning (9). Therefore, it is necessary to encourage older people to achieve SA with morality as the most convincing and effective indicator. Studies report that SA is associated with a low mortality rate, whereas the hazard ratio (HR) is inconsistent. A study from the United States shows that the lowest SA scorers had a 356% higher risk of mortality compared with individuals with the highest scores (10). Another study from China indicates that, compared with a non-successful aging (NSA) group, the death rate in the SA group decreased by 38% (11). To quantitatively figure out the mortality risk reduction effect of SA, a systematic review and meta-analysis of the studies researching the correlation of SA and mortality is needed. Currently, the correlation between SA and mortality has not been systemically examined. Therefore, this systematic review and meta-analysis is conducted to determine the impact of SA on all-cause mortality risk to provide a theoretical basis for promoting SA in older people, thereby improving the life quality of older people and reducing the family and society burden.

## Methods

### Search Strategy

The meta-analysis closely followed the recommendations of the preferred reporting items for systematic review and meta-analyses and the meta-analysis of observational studies in epidemiology (12, 13). A systematic search of three English databases (PubMed, Embase, and CINAHL) and two Chinese databases (CNKI and WanFang) was performed with a cutoff date at March 4, 2021, using the following keywords: [(successful aging OR healthy aging OR aging well) AND (mortality OR mortalities OR death OR fatal)] AND (risk OR Cox OR hazard OR survival analysis OR odds) for the English databases and Chinese translations of terms meaning SA and mortality for the Chinese databases (Supplementary Material 1 shows the detailed search strategy). References of selected articles were also searched to avoid missing related studies.

### Inclusion and Exclusion Criteria

Studies that met the following criteria were included: (1) study design: cohort study; (2) participants ≥ 60 years of age; (3) exploring the relationship between SA and mortality; (4) all-cause mortality risk was estimated using HR and 95% confidence intervals (CI); (5) SA was set as a dichotomous or continuous variable; (6) when SA was a dichotomous variable, all-cause mortality risk of either SA or NSA group was reported; and/or the risk of mortality from SA when SA was a continuous variable; and (7) full text published in English or Chinese.

Exclusion criteria were (1) duplicated articles; (2) case reports, reviews, meta-analysis, meeting or conference abstracts, comments or letters; (3) different articles using repetitive data (in this case, the most recently published article was selected.

### Data Extraction and Quality Assessment

According to the selection criteria, two trained authors independently and progressively screened the eligible articles by reading the title and abstract and then the full text. Any disagreements were resolved by discussion with the third author. After confirming the included studies, two reviewers independently extracted the following information from each research: first author, year of publication, population, country of origin, definition of SA, sample size and/or number of deaths, number of males and females, age, length of follow-up, HR, and 95% CI. If more than one model was used to evaluate the mortality risk of SA, the data (HR and 95% CI) of the model with the most adjusted variable was extracted. The Newcastle–Ottawa scale (NOS) for cohort studies (14) was used to assess the quality of eligible publications. The full scores is nine. Studies with NOS scores of ≥6 were considered of good quality.

### Data Analysis

STATA 16.0 was used for statistical analysis. The mortality HR and its 95% CI for SA were combined using a random-effects model as it was preferable and could provide wider CI than a fixed-effects model (15). *I*^2^ was used to assess interstudy heterogeneity, and *I*^2^ thresholds ≥25, ≥50, and ≥75% represented low, moderate, and high heterogeneity, respectively (16). Significant heterogeneity was present when *I*^2^ was more than 50%. Subgroup and sensitivity analyses were performed to evaluate the sources of heterogeneity. If the selected study reported the risk of death at a different length of follow-up, data on the longest follow-up interval was used to synthesize the overall mortality risk, and risk of mortality for different follow-up durations were used for subgroup analysis based on length of follow-up if not in the same subgroup. A funnel plot and Egger's test were combined to test publication bias when the number of studies included was not <10. The power of the test is too low to distinguish chance from real asymmetry if fewer than 10 studies are included (17). The statistical significance level was *P* < 0.05.

## Results

### Study Selection

**Figure 1:**
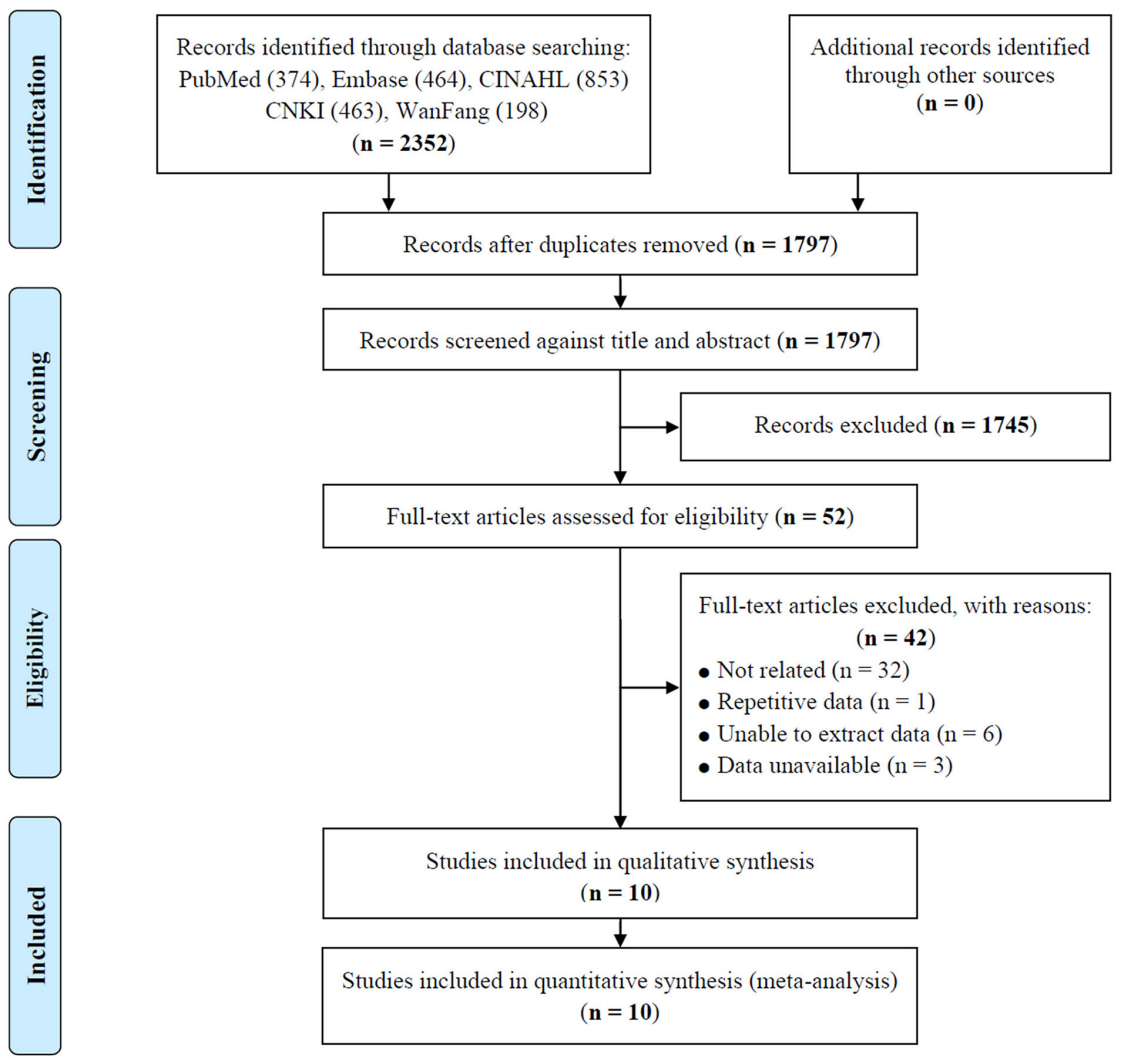
Flow diagram of study selection.

After screening by selection criteria, 10 studies investigating the effect of SA on all-cause mortality were included, which involved a total of 21,158 older adults. The selection process is shown in Figure 1.

### Study Characteristics

Baseline characteristics and definition of SA for each study included in the systematic review and meta-analysis are presented in Table 1. Among the 10 included cohort studies, 6 of them set SA as a dichotomous variable with total participants of 11,668, which involved 1974 SA older people and 9694 NSA older people (7, 11, 18, 19, 21, 22, 26). The other four articles (20, 23–25) set SA as a continuous variable and reported the HR (95% CI) when HAI was a four-category variable with 9,490 older adults included. Seven of the 10 studies took place in high-income countries and the others in upper-middle-income countries. According to NOS, all the 10 included articles were of high quality. The scoring details of the 10 articles are tabulated in Table 2.

**Table 1:** Characteristics of the included studies.

**References**	**Population**	**Country**	**Definition of SA**	**Sample Size** **(no. of death)**	**Sex** **(M/F)**	**Age** **(y)**	**Follow up** **(y)**	**Adjusted covariates**	**HR (95% CI)**	
Camozzato et al. (7)	The Porto Alegre Longitudinal Aging (PALA) study	Brazil	Very good state of health- a complete absence of functional disability and mood changes- and no cognitive impairment	Total: 234 (96) SA: 143 (46) NSA: 91 (50)	110/124	≧60 70.67 ± 6.74	12	Age, sex	M+F	0.53 (0.31–0.68)^*^
Kim et al. (18)	The Korean Longitudinal Study of Aging (2006–2014)	Korea	Participants met the four following factors; absence of the seven major diseases and the risk factors for these diseases, freedom from disability, high physical function, high cognitive function, and engagement in life.	Total: 2,960 SA: 139 NSA: 2821	1,285/1,675	≧65	7	Marital status, education, income, smoke, drink, physical activity	M: 64–74 y ≧75 y F: 64–74 y ≧75 y	0.19 (0.07–0.50)* 0.45 (0.17–1.20)* 0.10 (0.01–0.75)* 0.99 (0.44–2.22)^*^
Li et al. (19)	China Health and Retirement Longitudinal Survey (CHARLS) (2010–2016)	China	Being simultaneously without chronic disease, no functional loss, high cognitive function, no depression, and active participation in social activities	Total: 4,824 (136) SA: 746 (9) NSA: 4,078 (127)	2,417/2,407	≧60	1	Marital status, type of household registration, education, income, smoke, drink	M F	0.49 (0.20–1.24)* 0.37 (0.13–0.99)^*^
McCabe et al. (20)	The fifth examination cycle of Framingham Offspring Study (1991–1995)	USA	HAI (SBP, fasting glucose, creatinine, Mini-Mental State Exam, FVC) (0[healthiest]-10[unhealthiest])	Total: 934 (138)	460/474	≧60 65.6 ± 4.4	10	Age, sex, physical activity index, smoke, baseline cancer, BMI, hypertension, CVD, diabetes, kidney disease, and pulmonary disease	HAI 0–2 3–4 5–6 7–10	1.15 (1.00–1.34) 1 1.48 (0.89–2.48) 2.23 (1.13–4.40) 2.29 (0.89–5.88)
Negash et al. (21)	Community-dwelling non-demented adults (1986–2004)	USA	Patients with the mean of the four domain scores (memory, executive function, language, and visuo- spatial skills) in the top 10%.	Total: 560 SA: 56 NSA: 504	192/368	≧65 79.7 ± 6.5	7.9^a^	Age, sex, education, Charlson Index	M+F	0.70 (0.47–1.05)^*^
Nosraty et al. (22)	Two Vitality 90+ cross-sectional mailed surveys (2001 and 2003)	Finland	No disease, independent in five functional activities + no depressiveness, self-rated health average or better, willing to live up to 100 years + phone contacts, met children during past 2 weeks	Total: 1,370 Follow up 4 y: SA: 30(16) NSA: 1,340 (855) Follow up 7 y: SA: 30(20) NSA: 1,340 (1,126)	274/1096	≧90 91.8^b^	4 and 7	M+F: age, sex M/F: age	M+F (4 y) M (4 y) F (4 y) M+F (7 y) M (7 y) F (7 y)	0.71 (0.43–1.16)* 0.66 (0.27–1.60)* 0.72 (0.40–1.31)* 0.81 (0.53–1.21)* 0.66 (0.31–1.41)* 0.87 (0.54–1.40)^*^
O'Connell et al. (23)	The Health, Aging, and Body Composition (Health ABC) Study	USA	HAI (SBP, FVC, DSST, cystatin C, and fasting glucose) (0[healthiest]-10[unhealthiest])	Follow up 15.2 y Total: 2,264 (1,436) Follow up 6.2 y Total: 1,122 (497)	Follow up 15.2 y 1,100/1,164 Follow up 6.2 y 528/594	70–79 Follow up 15.2 y 73.6 ± 2.8 Follow up 6.2 y 82.1 ± 2.7	15.2^b^ and 6.2^b^	Age, sex, site, race, education, BMI, smoke, physical activity, cancer, CVD, pulmonary disease, depression, osteoporotic drugs, hip or knee osteoarthritis, gait speed	HAI(15.2 y) 0–2 3–4 5–6 7–10 HAI(6.2 y) 0–2 3–4 5–6 7–10	1.17 (1.13–1.21) 1 1.39 (1.13–1.71) 1.8 1 (1.46–2.24) 2.45 (1.94–3.08) 1.20 (1.14–1.27) 1 1.39 (0.80–2.40) 2.05 (1.21–3.47) 2.66 (1.57–4.52)
Sanders et al. (24)	Cardiovascular Health Study (CHS)	USA	HAI (SBP, FVC, creatinine, fasting glucose, Modified Mini-Mental Status Examination) (0[healthiest]-10[unhealthiest])	Total: 3,841 (2,242)	1,611/2,230	≧65 74.5 ± 5.0	12.8^a^	Age, sex, race, smoke, BMI, education, physical activity, baseline chronic conditions, incident coronary heart disease, and incident cerebrovascular disease	HAI 0–2 3–4 5–6 7–10	1.17 (1.14–1.21) 1 1.25 (1.09–1.44) 1.66 (1.44–1.91) 2.62 (2.22–3.10)
Shi et al. (11)	8 longevity areas of China: the Chinese Longitudinal Healthy Longevity Survey (CLHLS) (2012)	China	Having a score (based on 5 items: self-rated health, self-related psychological status, cognitive function, activities of daily life, physical activity) of more than 3 points.	Total: 1,720 (278) SA: 860(113) NSA: 860 (165)	846/874	≧65	2	Age, sex	M+F	0.62 (0.49–0.79)^*^
Wu et al. (25)	National Health and Nutrition Examination Survey (1999–2000, 2001–2002)	USA	mHAI (SBP, DSST, cystatin C, glucose, and respiratory problems) (0[healthiest]-10[unhealthiest])	Total: 2,451 (925)	1,064/1,387	≧60	9.1^b^	Age, sex, race, education, marital status, smoke, physical activity, BMI, and count of chronic conditions	mHAI 0–2 3–4 5–6 7–10	1.19 (1.11–1.27) 1 1.63 (1.07–2.50) 2.34 (1.56–3.50) 3.27 (2.05–5.24)

*Values represented as number, mean ± standard deviation, median^a^, mean^b^*.

* *NSA as the reference group*.

*SA, successful aging; NSA, non-successful aging; M, male; F, female; y, year; HR, hazard ratio; CI, confidence intervals; HAI, healthy aging index; mHAI, modified healthy aging index; SBP, systolic blood pressure, FVC, forced expiratory volume; BMI, body mass index; CVD, cardiovascular disease; DSST, digit symbol substitution test*.

**Table 2:** Results of the Newcastle-Ottawa scale quality assessment.

	**Selection** **(****4****)**				**Comparability (2)**	**Outcome** **(****3****)**			**Quality (9)**
	**Representativeness of the exposed cohort**	**Selection of the non-exposed cohort**	**Ascertainment of exposure**	**Demonstration that outcome of interest was not present at start of study**	**Comparability of cohorts on the basis of the design or analysis**	**Assessment of outcome**	**Was follow-up long enough for outcomes to occur**	**Adequacy of follow up of cohorts**	
Camozzato et al. (7)	1	1	1	1	0	0	1	1	6
Kim et al. (18)	1	1	1	1	1	1	1	0	7
Li et al. (19)	1	1	1	1	2	0	0	0	6
McCabe et al. (20)	1	1	1	1	0	1	1	1	7
Negash et al. (21)	1	1	1	0	2	1	1	0	7
Nosraty et al. (22)	1	1	1	1	1	1	1	1	8
O'Connell et al. (23)	1	1	1	1	0	1	1	0	6
Sanders et al. (24)	1	1	1	1	2	1	1	1	9
Shi et al. (11)	1	1	1	1	2	0	0	1	7
Wu et al. (25)	1	1	1	1	1	1	1	1	8

*A study can be awarded a maximum of one point for each numbered item within the Selection and Outcome categories. A maximum of two points can be given for Comparability. The total points are nine*.

### The Effect of SA on All-Cause Mortality Risk in Older Adults

**Figure 2:**
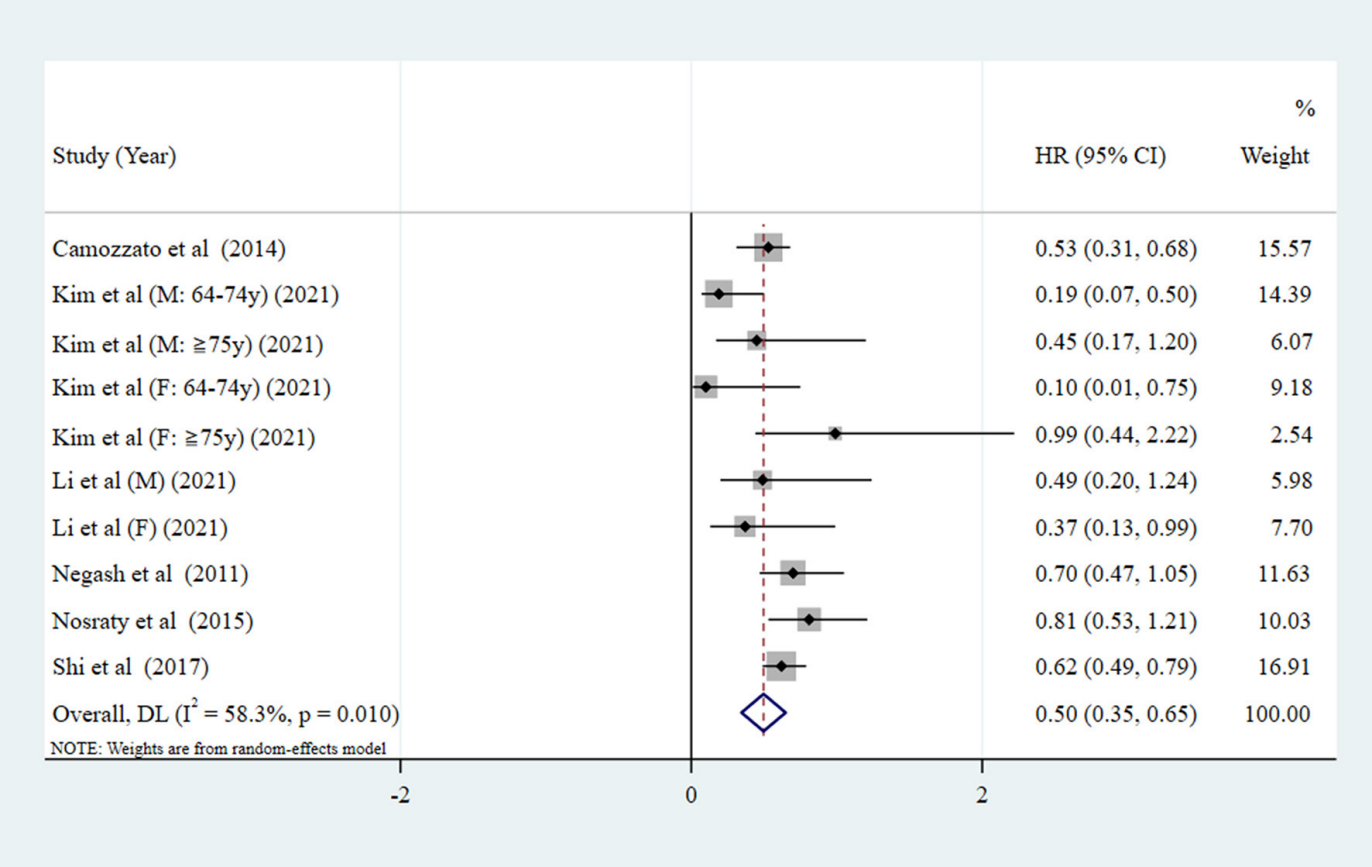
Forest plot of the pooled HR of all-cause mortality risk for SA (SA as a dichotomous variable. NSA as the reference group. SA, successful aging; NSA, non-successful aging; HR, hazard ratio).

**Figure 3:**
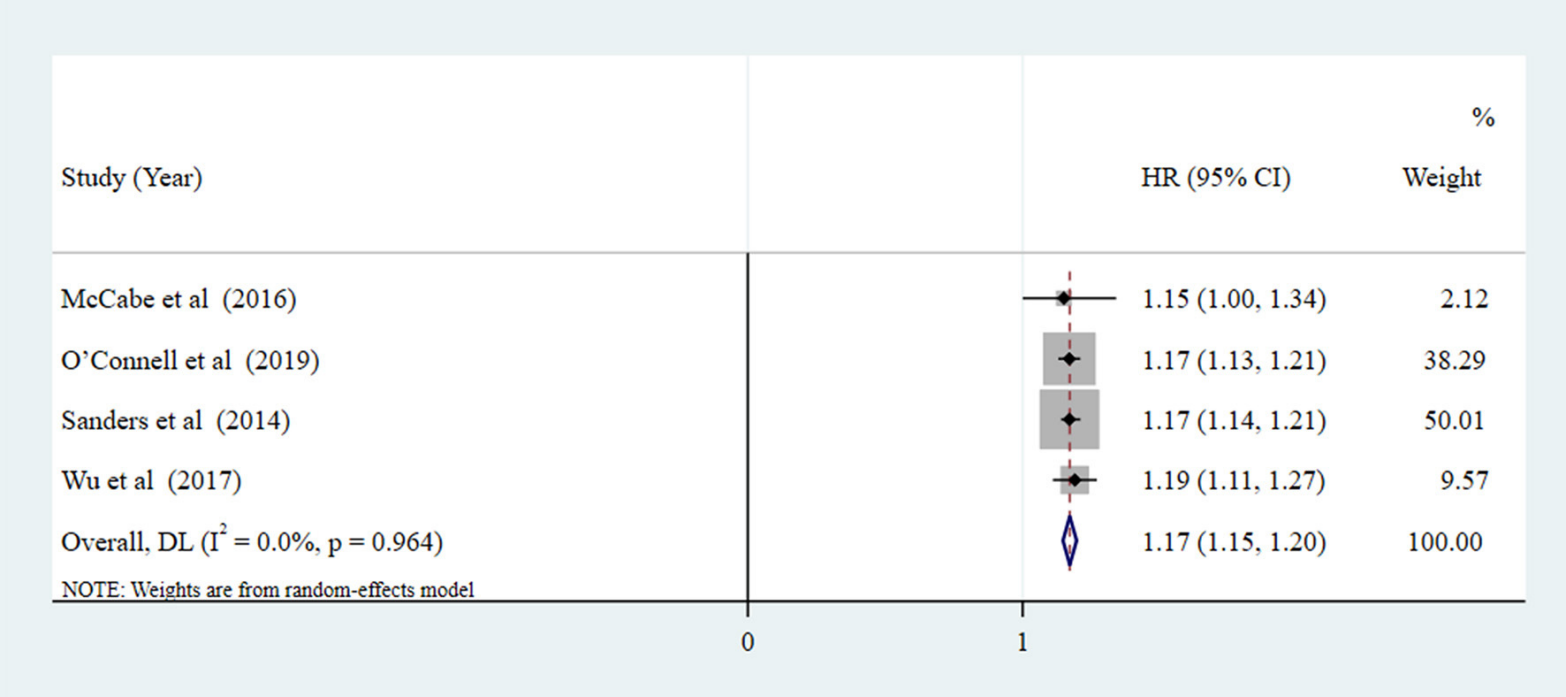
Forest plot of the pooled HR of all-cause mortality risk for SA (SA as a continuous variable, measured by HAI: The scores for each component were arranged into least healthy group (two points), middle group (one point), and most healthy group (zero points). The component scores were summed to create the HAI, ranging from 0 [healthiest] to 10 [unhealthiest]).

**Figure 4:**
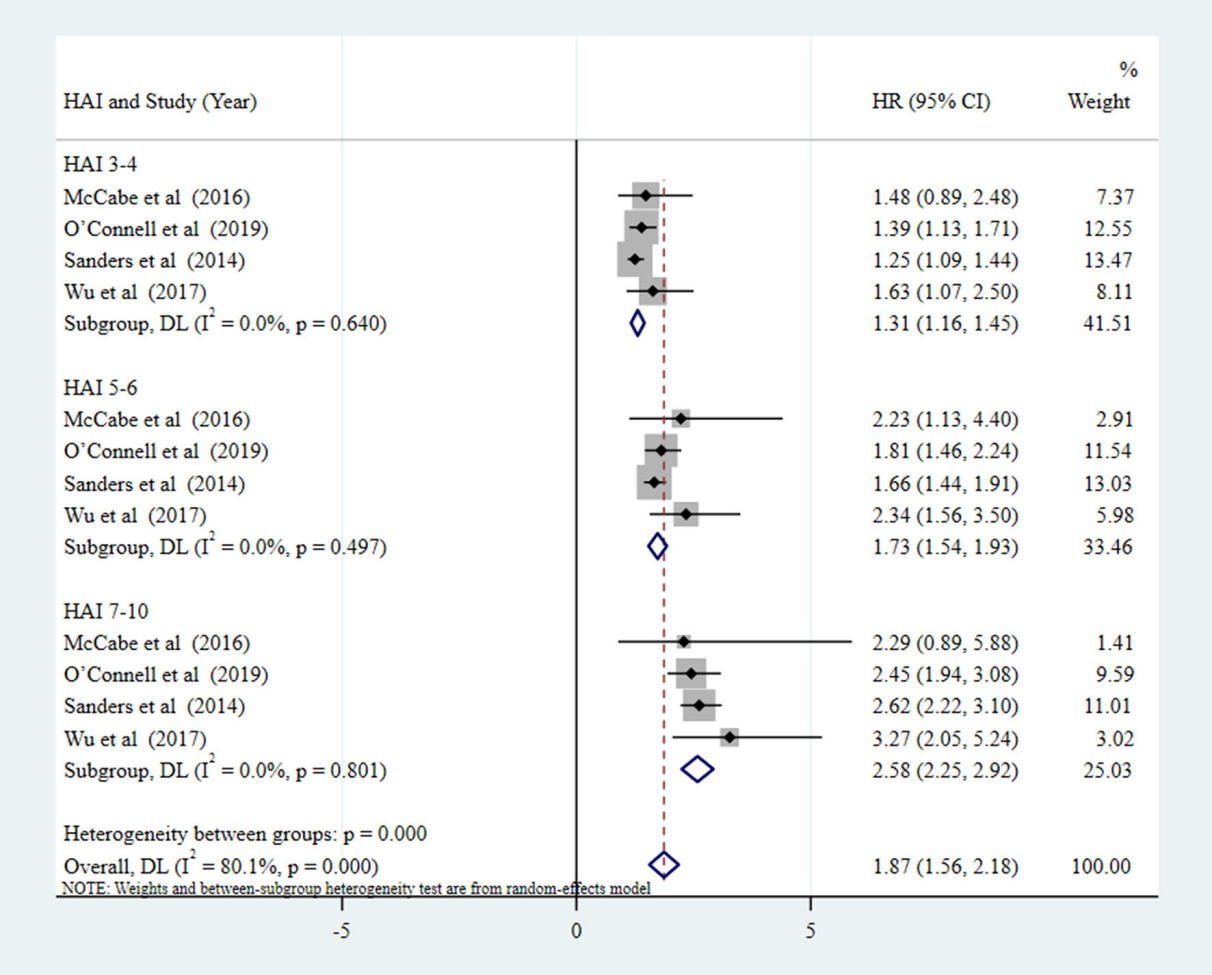
Forest plot of the pooled HR of all-cause mortality risk for SA (SA as four-category variable, measured by HAI: HAI 0-2 [healthy], HAI 3-4, HAI 5-6, HAI 7-10 [unhealthy], respectively. HAI 0-2 as the reference group).

Figure 2 indicates the risk of all-cause mortality when SA was a dichotomous variable (SA vs. NSA). The results show a 50% reduction in the risk of all-cause mortality in the SA group compared with the NSA group (pooled HR = 0.50, 95% CI: 0.35–0.65, *P* < 0.001; *I*^2^ = 58.3%). As presented by Figure 3, when SA was a continuous variable, the risk of all-cause mortality increased by 17% for each unit increment in HAI (*I*^2^ = 0%, *P* = 0.964). Compared with the reference group (HAI 0-2, healthy), older people with HAI 3-4, HAI 5-6, and HAI 7-10 had 1.31-fold, 1.73-fold, and 2.58-fold greater risk of all-cause mortality, respectively (Figure 4).

### Subgroup Analysis

To find the source of heterogeneity in the synthesis HR when SA is a dichotomous variable (Figure 2), a subgroup analysis was performed based on sex, length of follow-up, income, region, and year of publication. As shown in Table 3, none of those variables might be a source of heterogeneity. Notably, the risk of all-cause death was lower in the SA group than the NSA group in all subgroup analysis although the differences were not statistically significant. Compared with NSA group, the all-cause mortality risks in male SA and female SA were reduced by 66.7 and 48.6%, respectively. The <5 and >5-year mortality of SA older people were 39.9 and 51.2% lower than that of NSA, respectively. In addition, survival rates for SA older people in high-income countries tended to be higher than in upper-middle-income countries, and those from Asia were higher than the others. Moreover, articles published in 2016–2021 reported a lower risk of all-cause death among SA older people than in 2011–2015.

**Table 3:** Summary estimates on the risk of mortality between subgroups.

**Subgroups**	**No. of studies**	**Hazard ratio**	**95% confidence intervals**		***P***	**Heterogeneity**		**Q (*P*)**
			**Lower**	**Upper**		***I*^2^ (%)**	***P***	
Sex								0.59 (0.442)
Male	3	0.333	0.125	0.542	0.002	14.0	0.322	
Female	3	0.514	0.104	0.924	0.014	65.3	0.034	
Length of Follow up								0.79 (0.373)
> 5 years	4	0.488	0.274	0.702	<0.001	67.2	0.006	
<5 years	3	0.601	0.473	0.729	<0.001	0	0.640	
Income								0.27(0.604)
Upper middle income	3	0.566	0.456	0.676	<0.001	0	0.677	
High income	3	0.486	0.205	0.767	0.001	71.4	0.004	
Region								2.82 (0.093)
Asia	3	0.402	0.189	0.615	<0.001	61.4	0.016	
non-Asia	3	0.631	0.470	0.792	<0.001	16.7	0.301	
Publication Year								2.82 (0.093)
2011–2015	3	0.631	0.470	0.792	<0.001	16.7	0.301	
2016–2021	3	0.402	0.189	0.615	<0.001	61.4	0.016	

### Sensitivity Analysis and Publication Bias

Sensitivity analysis indicates that all the estimated values were in regions of the lower and upper CI limit, which revealed that removing any single study did not alter the overall results (figure not shown). Egger's test was not performed to test publication bias because the number of studies using SA as a dichotomous variable or as a continuous variable were both fewer than 10. However, we did a funnel plot to visually display the research offset of literature and found that it did exist (Supplementary Material 2).

## Discussion

The world population has been aging rapidly. The proportion of the world population aged 60 years or older increased from 8% in 1950 to 12% in 2013 (27). Population aging is shifting the disease pattern to chronic diseases and disabilities with more complex health conditions, which exerts greater pressures on the health system and increased costs (28). SA has been a goal in the field of gerontology for a long time and is of great interest to researchers in the field of public health (27, 29). Studies report that achieving SA would result in a significant mortality rate reduction (7, 11, 18, 22), which is confirmed in this systematic review and meta-analysis of 10 studies involving 21,158 older people. The results show that the SA group tended to have 50% lower risk of all-cause mortality than the NSA group. In addition, all-cause death risk increased by 17% for each unit increment in HAI. Compared with the HAI 0-2 group, older people with HAI 3-4, HAI 5-6, and HAI 7-10 had 1.31-fold, 1.73-fold, and 2.58-fold greater risk of all-cause mortality, respectively. Therefore, it is necessary for health workers to intervene and help older adults achieve SA. To our knowledge, this meta-analysis is the first to seek the impact of SA on the risk of all-cause mortality in older adults.

Subgroup analysis found that none of the sex, length of follow-up, income, region, and year of publication factors might be a source of heterogeneity. No statistical difference between the groups was found. However, the results showed that, compared with NSA group, the risk of all-cause mortality in male SA reduced by 66.7%, and the value was 48.6% in females, which was consistent with the results reported by Kim et al. (18) and Nosraty et al. (22). Whereas, the reasons for the gender differences are not clear, combined with existing research, the gender differences in mortality rates are largely attributable to gender differences in social relationships, dysfunction, socioeconomic status, health behaviors, biomarkers, and genetic variations (30–35). In this meta-analysis, the risk of <5 and >5-year all-cause mortality in older adults with SA were all lower than that in the NSA population, which implies that older people who achieved SA lived longer than those who did not. The possible reason is that, although the aging process is dynamic and these successful agers may experience some degree of decline, the SA process means delaying the onset of disability, compressing morbidity, or shortening the proportion of life expectancy for disability, and this resilience may confer a higher longevity (7, 36).

We also found that non-Asians who had successfully aged had a higher risk of death than their Asian counterparts (63.1 vs. 40.2%). It is confirmed that a healthier diet according to the World Health Organization guidelines is associated with lower risk of death (37). The Western diet is characterized by being higher in total fat, saturated and trans fatty acids, and keys dietary lipid score, lower in total carbohydrate and starch, and higher in sugars (38), which relates to higher average levels of serum total cholesterol and higher mortality from coronary heart disease in Western than East Asian populations (38). In addition, survival rates for SA older people in high-income countries tended to be higher than in upper-middle-income countries. It is reported that, compared with high-income countries, middle-income countries had higher rates of household air pollution, poor diet, low education, and low grip strength, which were related to mortality (39). Therefore, these aspects need to be given sufficient attention.

One interesting finding is that articles published in 2016–2021 reported a lower risk of all-cause death risk among SA older people than in 2011–2015 (42.2 vs. 63.1%) although there was no significant difference between groups. It may be that with SA being more widely mentioned in the past 5 years, people who have entered the old age pay more and more attention to maintenance and health preservation. They know that SA state is not unalterable and needs to be maintained with effort. Even if the current state is well, older adults still maintain a good healthy lifestyle and mental state. Among older people who have not aged successfully, they may be worried about some abnormality in the body, resulting in anxiety, depression, and other negative emotions, which are associated with increased all-cause mortality (40, 41).

However, there are some shortcomings in this systematic review and meta-analysis. First, although subgroup and sensitivity analyses were conducted, possible sources of heterogeneity are still not identified. Second, because there were no studies from low-income countries that met the inclusion criteria, the generalization of this study in these countries needs to be confirmed by further studies. Third, due to the limited number of articles included, a funnel plot and Egger's test were not used to test publication bias. Therefore, more relevant studies are needed to verify the results of this meta-analysis in the future.

## Conclusion

This meta-analysis suggests that SA of older adults can reduce all-cause mortality risk. However, few studies have concentrated on interventions to help older people achieve SA. Therefore, healthcare providers must be aware of the relationship between SA and mortality risk and be actively involved in developing intervention methods for helping old people achieve SA.

## Data Availability Statement

The original contributions presented in the study are included in the article/Supplementary Material, further inquiries can be directed to the corresponding authors.

## Author Contributions

LM and RY formulated the research question, designed this review, screened the articles, analyzed the data, and drafted the manuscript. JC screened the articles, checked the extracted data, and verified the results of data analysis. MN and LX extracted the data. WS and XS assisted with checking the rationality of the design and supervising the quality of the manuscript. All authors approved the final version of the manuscript.

## Funding

This work was supported by grants from Science, Education and Health, Youth Science and Technology Project of Suzhou City (RY, No. KJXW2018007); Geriatric Health Research Project of Jiangsu Province (XS, No.LK2021018).

## Conflict of Interest

The authors declare that the research was conducted in the absence of any commercial or financial relationships that could be construed as a potential conflict of interest.

## Publisher's Note

All claims expressed in this article are solely those of the authors and do not necessarily represent those of their affiliated organizations, or those of the publisher, the editors and the reviewers. Any product that may be evaluated in this article, or claim that may be made by its manufacturer, is not guaranteed or endorsed by the publisher.
