# Supplementary material for: The Relationship Between Successful Aging and All-Cause Mortality Risk in Older Adults: A Systematic Review and Meta-Analysis of Cohort Studies
Source: Front Med (Lausanne). 2022 Feb 9;8:740559. doi: 10.3389/fmed.2021.740559 (PMC8864313; doi:10.3389/fmed.2021.740559)
Supplement: Supplementary file 1 [file Data_Sheet_1.DOC]

**Supplementary 1** Search strategy

- **English databases**

**Keywords:** ((successful aging OR healthy aging OR aging well) AND (mortality OR mortalities OR death OR fatal)) AND (risk OR Cox OR hazard OR survival analysis OR odds)

**Pubmed: 374**

**#1** ((successful aging[Title/Abstract]) OR (healthy aging[Title/Abstract])) OR (aging well[Title/Abstract])

**#2** (((mortality) OR (mortalities)) OR (death)) OR (fatal)

**#3** ((((risk) OR (Cox)) OR (hazard)) OR (survival analysis)) OR (odds)

**#4** English[Language]

**#5 #1** AND **#2** AND **#3** AND **#4**

**Embase: 464**

**#1** 'successful aging':ab,ti OR 'healthy aging':ab,ti OR 'aging well':ab,ti

**#2** mortality OR mortalities OR death OR fatal

**#3** risk OR cox OR hazard OR (survival AND analysis) OR odds

**#4** [humans]/lim AND [english]/lim

**#5 #1** AND **#2** AND **#3** AND **#4**

## CINAHL Complete: 853

**#1** TI successful aging OR AB successful aging OR TI healthy aging OR AB healthy aging OR TI aging well OR AB aging well

**#2** mortality OR mortalities OR death OR fatal

**#3** risk OR Cox OR hazard OR survival analysis OR odds

**#4** LA english

**#5 #1** AND **#2** AND **#3** AND **#4**

- **Chinese databases**

**Wangfang**: **198**

检索表达式： 题名或关键词:("成功老龄化") or 题名或关键词:("健康老龄化") and 全部:("死亡")

**CNKI: 463**

（主题：成功老龄化）OR（主题：健康老龄化）AND（全文：死亡）
