# Supplementary material for: The Relationship Between Successful Aging and All-Cause Mortality Risk in Older Adults: A Systematic Review and Meta-Analysis of Cohort Studies
Source: Front Med (Lausanne). 2022 Feb 9;8:740559. doi: 10.3389/fmed.2021.740559 (PMC8864313; doi:10.3389/fmed.2021.740559)
Supplement: Supplementary file 2 [file Data_Sheet_2.DOCX]

**Supplementary 2** Funnel plot


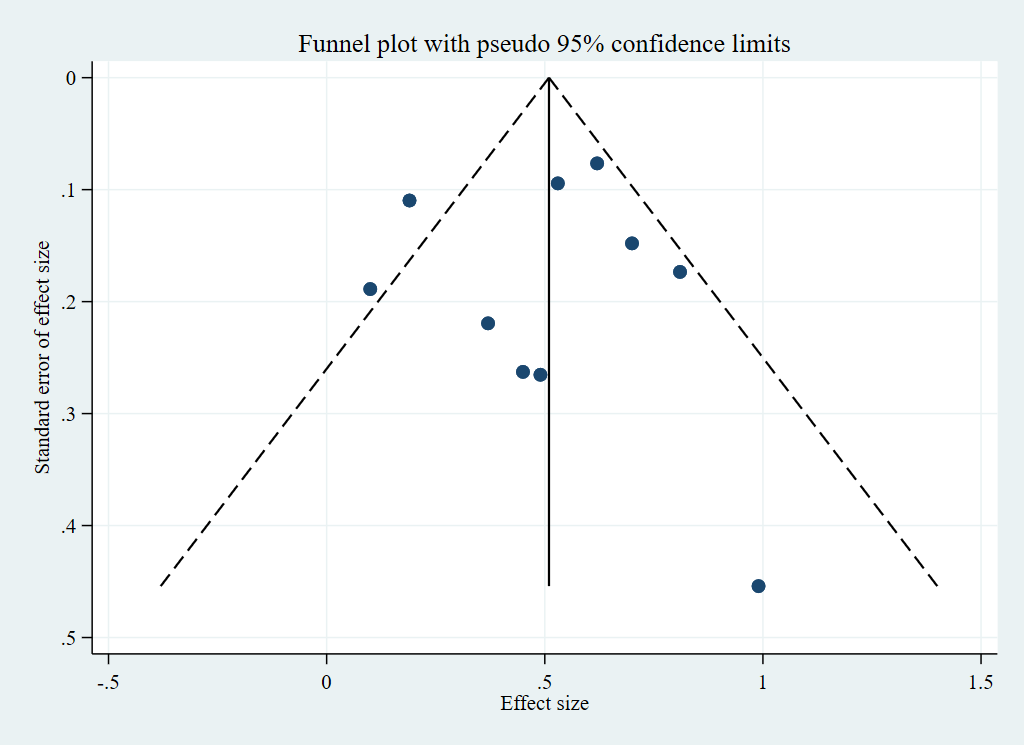


**Figure S2.1** Funnel plot of the effect of SA on all-cause mortality risk in older adults (SA as dichotomous variable)


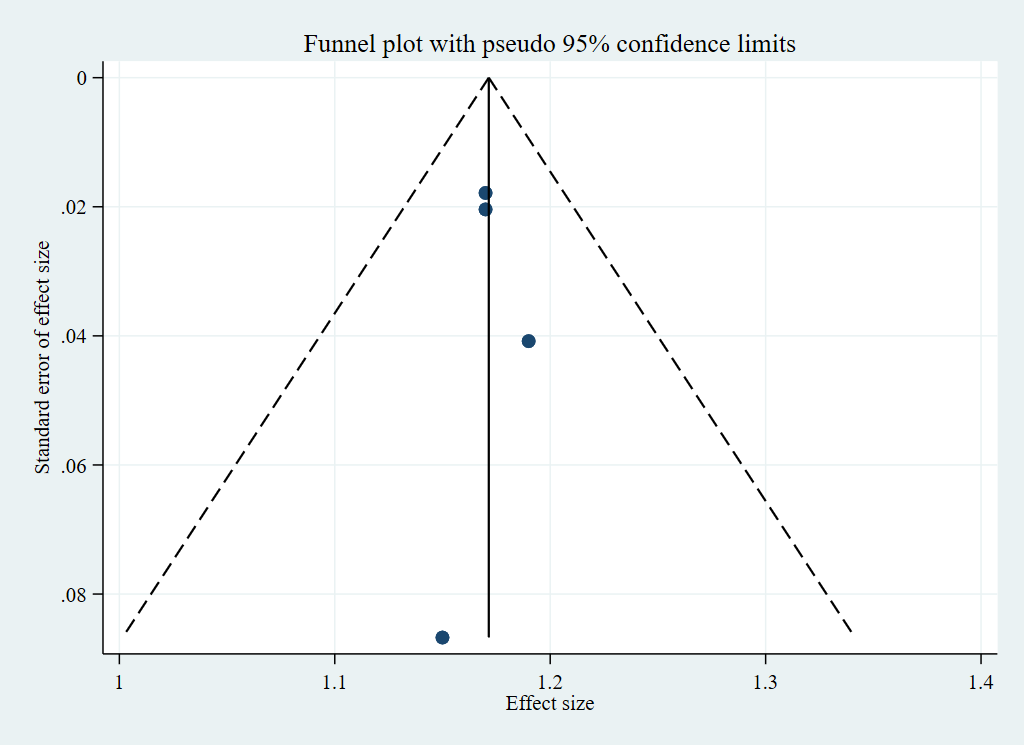


**Figure S2.2** Funnel plot of the effect of SA on all-cause mortality risk in older adults (SA as continuous variable)
